# Prognostic and Predictive Significance of B7-H3 and CD155 Expression in Gastric Cancer Patients

**DOI:** 10.3390/diagnostics15212695

**Published:** 2025-10-24

**Authors:** Ozlem Dalda, Zehra Bozdag, Sami Akbulut, Hasan Gokce, Yasin Dalda, Ayse Nur Akatli, Mustafa Huz

**Affiliations:** 1 Department of Pathology, Faculty of Medicine, Inonu University, 44280 Malatya, Türkiye; 2 Department of Surgery, Liver Transplant Institute, Faculty of Medicine, Inonu University, 44280 Malatya, Türkiye; 3 Department of Biostatistics and Medical Informatics, Faculty of Medicine, Inonu University, 44280 Malatya, Türkiye

**Keywords:** gastric adenocarcinoma, B7-H3 stain, CD155 stain, RT-PCR, prognostic biomarker, metastasis, lymphovascular invasion, perineural invasion

## Abstract

**Background/Objectives**: This study aimed to characterize the expression patterns of B7 homolog 3 (B7-H3) and cluster of differentiation 155 (CD155), two immune-related transmembrane glycoproteins, in resectable gastric adenocarcinoma and to elucidate their clinicopathological, prognostic, and molecular implications. **Methods**: The study included 112 patients who underwent gastrectomy for gastric adenocarcinoma between 2020 and 2025, along with 30 samples of normal gastric tissue obtained from sleeve gastrectomy specimens. Histological subtype, grade of differentiation, TNM stage, and invasion parameters were re-evaluated. Immunohistochemical expression of B7-H3 and CD155 was quantified for membranous, stromal and membranous/cytoplasmic staining patterns. Quantitative reverse transcription polymerase chain reaction (RT-PCR) was performed on 29 tumor and 25 normal samples to confirm mRNA expression levels, with fold change ≥2 considered biologically significant upregulation and ≤0.5 considered downregulation. Machine learning models were developed to predict metastasis and mortality based on clinical and immunohistochemical features. **Results**: 78.5% of tumors were at an advanced stage (T3–T4), and metastasis was present in 22.3% of patients. Perineural invasion (PNI) and lymphovascular invasion (LVI) were observed in 67.9% and 88.4% of cases, respectively. Increased B7-H3 and CD155 expression were significantly associated with advanced tumor stage, metastasis, and the presence of PNI and LVI (all *p* < 0.05). In metastatic tumors, median membranous B7-H3, stromal B7-H3, and CD155 scores were 60, 130, and 190, respectively, compared with 20, 90, and 120 in non-metastatic tumors. A significant positive correlation was found between stromal B7-H3 and CD155 expression (r = 0.384, *p* < 0.001), indicating parallel upregulation. Quantitative RT-PCR confirmed significant overexpression of both genes in tumor tissues relative to normal controls. B7-H3 was upregulated in 75.9% and CD155 in 58.6% of samples, with co-upregulation in 55.2%. Fold-change levels were markedly higher in metastatic versus non-metastatic cases (B7-H3: 7.69-fold vs. 3.04-fold; CD155: 7.44-fold vs. 1.79-fold). ML analysis using the XGBoost model achieved 91.1% accuracy for metastasis prediction (F1-score 0.800). Key variables included pathological T4b stage, perineural invasion, N3b status, T4a stage, and CD155 score. The mortality model yielded 86.7% accuracy (F1-score 0.864), with metastasis, differentiation status, nodal involvement, age, lymph node ratio, and perineural invasion emerging as principal predictors. **Conclusions**: Combined evaluation of B7-H3 and CD155, supported by immunohistochemical staining and RT-PCR quantification of B7-H3 and CD155 mRNA expression levels, provides meaningful prognostic insights and supports their potential as dual molecular biomarkers for aggressive gastric adenocarcinoma phenotypes.

## 1. Introduction

Gastric cancer is one of the most common malignant tumors of the gastrointestinal tract. According to GLOBOCAN 2022 data, it ranks fifth in global incidence, with 968,350 new cases, and fifth in cancer-related mortality, with 659,853 deaths. Despite significant advances in surgical techniques and systemic treatments, the prognosis of advanced gastric cancer remains poor, with median overall survival still below 12 months [1,2,3]. Several clinicopathological parameters—such as tumor size, depth of invasion, lymphovascular invasion (LVI) and perineural invasion (PNI), lymph node involvement, histological subtype, patient sex, and surgical margin status—have been identified as key determinants of mortality, metastatic potential, and overall tumor biology [4,5]. Building upon these established predictors, molecular classifications have refined the understanding of tumor behavior and improved the precision of therapeutic response prediction [6,7,8].

Recent advances in molecular oncology have revealed the crucial role of tumor–immune interactions in gastric carcinogenesis. As part of this evolving understanding, immunotherapy has emerged as a novel treatment paradigm for gastric cancer. Immune checkpoint blockade therapy targeting programmed cell death protein-1 (PD-1), programmed death-ligand 1 (PD-L1), and cytotoxic T-lymphocyte-associated protein 4 (CTLA-4) has emerged as a promising treatment option for gastric cancer. However, only a limited subset of patients achieves durable clinical responses to anti–PD-1, PD-L1, or CTLA-4 therapy [9]. This underscores the need to identify additional immune regulatory molecules involved in tumor immune evasion.

B7 homolog 3 protein (B7-H3, CD276) is an immune checkpoint molecule with dual co-stimulatory and co-inhibitory functions [10]. It was first cloned in 2001 from human dendritic cells [11]. Two isoforms have been identified: 4Ig-B7-H3, which predominates in malignant tissues, and 2Ig-B7-H3 [12,13]. Soluble B7-H3 (sB7-H3) has also been detected in serum, generated through proteolytic cleavage or alternative splicing [14]. Although its receptor has not been definitively identified, B7-H3 is known to modulate immune responses through interactions with T lymphocytes, antigen-presenting cells, and tumor cells [15]. It influences immune regulation and tumor progression through both immune and nonimmune mechanisms, enhancing tumor proliferation, invasion, migration, and resistance to apoptosis [16,17,18,19,20,21,22,23]. Overexpression of B7-H3 is consistently associated with poor prognosis in many malignancies [24].

Cluster of differentiation 155 (CD155, also known as poliovirus receptor, PVR) is a type I transmembrane glycoprotein of the immunoglobulin superfamily, acting as an adhesion molecule and immune modulator [25]. Normally expressed at low levels, CD155 is upregulated in tumor tissues, promoting cell proliferation, migration, and angiogenesis [26,27,28,29]. Its immunoregulatory role depends on receptor interactions with TIGIT, CD96, and DNAM-1/CD226. Binding of CD155 to TIGIT or CD96 transmits inhibitory signals that suppress T- and NK-cell activity, whereas interaction with DNAM-1 provides co-stimulatory signaling [25,30]. The TIGIT–CD155 axis therefore plays a central role in tumor immune escape, and its overexpression has been linked to poor prognosis in numerous cancers [31,32,33,34,35].

Recent advances in tumor immunology have emphasized the significance of immune-regulatory molecules within the tumor microenvironment. B7-H3 suppresses T-cell activity and contributes to nonimmune processes such as proliferation, invasion, metabolic reprogramming, and therapeutic resistance, while CD155 facilitates immune evasion through interaction with the TIGIT, DNAM-1, and CD96 receptors. High expression levels of both molecules promote tumor progression and correlate with poor survival outcomes [10,11,17,36,37].

Both B7-H3 and CD155 are immune checkpoint molecules that act through distinct yet complementary pathways to promote tumor immune evasion. B7-H3 primarily suppresses T-cell-mediated cytotoxicity, while CD155 interacts with receptors such as TIGIT and CD96 to inhibit antitumor T- and NK-cell activity [38,39,40,41]. Preclinical and interactome studies have suggested functional links between B7-H3 and CD155 signaling pathways, supporting the hypothesis that co-dysregulation of these molecules may contribute to immune evasion; however, large clinical studies directly assessing their co-expression and prognostic impact in gastric cancer are lacking [42,43,44]. Accordingly, simultaneous evaluation of these two markers may provide synergistic prognostic insights and a more comprehensive understanding of the immune landscape of gastric adenocarcinoma. This dual-marker approach aligns with previous multi-checkpoint studies—such as PD-L1/TIGIT and PD-L1/TIM-3 combinations—that have demonstrated clinically meaningful associations. To our knowledge, no previous study has examined the combined expression of B7-H3 and CD155 in gastric cancer.

This study expands the emerging evidence base on precision oncology by highlighting the immunological relevance of B7-H3 and CD155-mediated checkpoint pathways in gastric cancer. Beyond conventional clinicopathological indicators, the combined assessment of these biomarkers appears to refine risk stratification and uncover actionable prognostic and therapeutic axes. Notably, this is one of the earliest studies to investigate the co-expression dynamics of B7-H3 and CD155 in gastric cancer, underscoring their translational potential as dual immune biomarkers.

## 2. Materials and Methods

### 2.1. Study Period and Patient Selection

This study included a total of 112 patients diagnosed with gastric cancer who underwent surgical resection between 2020 and 2025 (study group) and 30 patients who underwent sleeve gastrectomy for non-malignant indications (control group). Patients in the study group who received preoperative neoadjuvant therapy were excluded. In addition, quantitative reverse transcription polymerase chain reaction (RT-PCR) was performed in 25 normal and 29 tumor tissue samples; fold change values >2 were considered significant upregulation, while values <0.5 indicated downregulation.

For all gastric cancer patients, demographic, clinical, and pathological data were retrospectively collected from the Hospital Information Management System and pathology archives. The variables assessed included age, sex, date of initial diagnosis, recurrence date (if applicable), tumor size, tumor volume, anatomical localization, histological grade, LVI, PNI, number of dissected lymph nodes, number of metastatic lymph nodes, depth of invasion (pathological T stage, pT), lymph node involvement (pathological N stage, pN), and presence of distant metastasis (M). Tumor stages were grouped as early stage (pT1–pT2) and advanced stage (pT3–pT4). All histopathological evaluations were performed independently by two experienced pathologists blinded to the clinical outcomes. Discrepancies were resolved by consensus.

### 2.2. Immunohistochemistry Protocol for B7-H3 and CD155 Staining

From the tumor blocks designated for immunohistochemical analysis, the most representative blocks with the highest tumor density and optimal fixation were selected. Immunohistochemical staining was performed using the Dako Omnis fully automated system. For B7-H3, a recombinant rabbit monoclonal IgG antibody (HUABIO, Woburn, MA, USA), clone: HA721245; dilution 1:5000) was applied, while for CD155, a recombinant rabbit monoclonal IgG antibody (Proteintech, Rosemont, IL, USA, clone: 241405F5; dilution 1:1500) was used. For B7-H3 membranous staining, prostatic acinar adenocarcinoma tissue was used as a positive control, and for stromal staining, lung adenocarcinoma tissue served as control. For CD155, liver tissue and colonic adenocarcinoma were used as positive controls.

In tumor samples, tumoral membranous and cytoplasmic staining of B7-H3 was evaluated separately from stromal staining, and each was scored independently. For CD155, both membranous and cytoplasmic staining (membranous/cytoplasmic) were evaluated and scored. All staining patterns of B7-H3 and CD155 were quantified using the histo-score (H-score) method. Staining intensity was graded as 0 (negative), 1 (weak), 2 (moderate), or 3 (strong) [45]. The H-score was calculated by multiplying the percentage of positively stained cells at each intensity by the corresponding intensity value and summing the results. The formula was as follows: H-score = (% of cells with intensity 0 × 0) + (% with intensity 1 × 1) + (% with intensity 2 × 2) + (% with intensity 3 × 3). The expression levels of B7-H3 and CD155 were then correlated with demographic, clinical, and pathological prognostic factors, including tumor stage, histologic grade, LVI, PNI, and lymph node metastasis.

### 2.3. Gene Expression Analysis Using Molecular Methods

To investigate the relative expression levels of CD155 and B7-H3, a total of 25 histopathologically normal control samples and 29 gastric cancer tissue samples were analyzed using quantitative gene expression profiling. Gastric cancer specimens were randomly selected from patients who underwent surgery between 2023 and 2025. Among the 30 patients in the gastric cancer cohort, 29 yielded tissue with sufficient RNA quality for mRNA isolation, while 25 out of 30 control samples retained intact messenger RNA suitable for downstream analysis.

Human beta-actin (ACTB) was used as the endogenous housekeeping gene to normalize target gene expression. Primer design for both reference and target genes was performed using the Qiagen GeneGlobe database, and commercially available Qiagen RT^2^ qPCR Primer Assays were utilized. Validated Primer Assays for human RNA expression analysis included: ACTB (GeneGlobe ID: PPH00073G, Cat. No: 330001), B7-H3 (GeneGlobe ID: PPH00736F, Cat. No: 330001), and CD155 (GeneGlobe ID: PPH10083A, Cat. No: 330001). Relative gene expression levels were quantified using the 2^−ΔΔCt^ method, also known as the Livak method [46,47]. In this calculation, fold change (2^−ΔΔCt^) represents the ratio of normalized gene expression in the test sample (2^−ΔCt^) to normalized gene expression in the control sample (2^−ΔCt^). A fold change greater than 1 was considered biologically meaningful upregulation, with a threshold of ≥2.0 indicating moderate to high-level upregulation. Conversely, a fold change ≤0.5 was interpreted as biologically significant downregulation.

### 2.4. Study Protocol, Ethical Approval, and Funding

This study was conducted in accordance with the principles of the Declaration of Helsinki (1964) and its subsequent amendments, as well as institutional and national ethical standards for research involving human participants. Approval for the study was obtained from the Inonu University Institutional Review Board (IRB) for Non-Interventional Studies (Approval Date: 30 July 2024; Approval No: 6205). Because the study had a retrospective design involving file review and re-evaluation of archived pathology slides, the requirement for individual informed consent was waived by the ethics committee. The research was financially supported by the Inonu University Scientific Research Projects Coordination Unit (Grant No: TTU-2024-3753). The design, conduct, analysis, and reporting of the study adhered to the Strengthening the Reporting of Observational Studies in Epidemiology (STROBE) guidelines to ensure methodological rigor and minimize potential sources of bias.

### 2.5. Statistical Data Analysis

All statistical analyses were conducted using SPSS software (version 2025; Statistical Package for the Social Sciences, IBM Corp., Armonk, NY, USA). Categorical variables were expressed as frequencies and percentages, while continuous variables were summarized as medians with 95% confidence intervals [median (95% CI)]. Normality of distribution was assessed using the Kolmogorov–Smirnov test. Since the variables did not follow a normal distribution, non-parametric tests were applied. Comparisons between two groups were conducted using the Mann–Whitney U test, and comparisons among three or more groups were performed with the Kruskal–Wallis H test. Categorical variables were compared using the Pearson chi-square test, chi-square test with Yates’ correction, or Fisher’s exact test, as appropriate. Survival analyses were carried out using the Kaplan–Meier method, and intergroup comparisons were assessed with the log-rank test. The relationships between immunohistochemical staining scores were examined using Spearman’s correlation analysis, which was interpreted as negligible (0.00–0.20), weak (0.21–0.40), moderate (0.41–0.60), strong (0.61–0.80), or very strong (0.81–1.00). A *p*-value <0.05 was considered statistically significant level. For each subgroup of the study, separate post hoc power analyses were conducted using G*Power version 3.1.9.6, confirming that all analyses had adequate statistical power.

### 2.6. Machine Learning Models for Tumor Behavior Predictions

For artificial intelligence modeling, data from 112 patients with gastric cancer were analyzed. The dataset included 26 variables encompassing demographic information, tumor characteristics, pathological findings, and molecular markers. To enhance data quality and reduce dimensionality, variable selection was performed using the Least Absolute Shrinkage and Selection Operator (LASSO) regression method. Data were standardized with the StandardScaler function, and 5-fold cross-validation was applied. Variables with non-zero coefficients after LASSO regression were retained for subsequent modeling. Categorical variables (sex, tumor differentiation, Lauren classification, WHO classification, clinical M stage, pathological T stage, pathological N stage, and TNM staging) were transformed using the one-hot encoding method, allowing the independent evaluation of each categorical level. To overcome the limitations of small sample size, bootstrap resampling was performed. The original dataset was resampled and expanded to 224 observations, thereby reducing the risk of overfitting and improving the generalizability of the model.

The Extreme Gradient Boosting (XGBoost) algorithm was selected as the classification model. The model parameters were set as follows: n_estimators = 100, max_depth = 6, and learning_rate = 0.1. The dataset was split into training (80%) and test (20%) subsets using stratified sampling to preserve class distribution across both sets. Model performance was evaluated using accuracy, sensitivity, specificity, positive predictive value (PPV), negative predictive value (NPV), and F1-score. Variable importance was assessed through the XGBoost feature importance function, and the ten most influential variables were visualized graphically. All analyses were performed using Python, employing the pandas library for data manipulation, numpy for numerical computations, and scikit-learn together with XGBoost for machine learning applications.

## 3. Results

### 3.1. General Assessment of the Entire Cohort

The study cohort consisted of 112 patients (median age, 65 years), with males comprising the majority (71.4%). The distal stomach was the most common tumor site (49.1%), and total gastrectomy was the predominant surgical procedure (50.9%). According to the Lauren classification, intestinal-type carcinoma was the most frequent histological subtype (59.8%), followed by poorly cohesive (21.4%) and mixed types (18.8%). PNI and LVI were identified in 67.9% and 88.4% of cases, respectively.

The median tumor diameter was 56 mm, corresponding to a median tumor volume of 60 mm^3^. A median of 30 lymph nodes were retrieved, 7 of which were positive, resulting in a median lymph node ratio of 25%. Regarding pathological staging, advanced primary tumors (T3–T4) accounted for 78.5% of cases, and nodal involvement was frequent, with half of the patients classified as N3a/N3b. Metastatic disease (M1) was present in 22.3% of patients. Consequently, most cases (67.9%) were classified as stage III or IV according to the TNM system.

The median membranous B7-H3 expression score was 30, indicating moderate expression, while stromal B7-H3 expression was markedly higher, with a median score of 100, reflecting strong staining in the tumor microenvironment. Membranous/cytoplasmic CD155 expression showed the highest levels among the evaluated biomarkers, with a median score of 140.

The median overall follow-up was 615 days, and the median disease-free survival was 542 days. At the final follow-up, 51.8% of patients had died, and metastasis and/or locoregional recurrence occurred in 29.5% of cases. Detailed clinicopathological and immunohistochemical data are summarized in Table 1, Supplementary Table S1, and Supplementary Figures S1 and S2. A post hoc power analysis using the *t* test (two-tailed test, effect size = 0.5, α = 0.05) demonstrated a statistical power of 0.999, confirming that the sample size of 112 patients was sufficient for reliable inference.

### 3.2. Comparison of Gastric Cancer Patients Based on Tumor Differentiation Characteristics

Tumor diameter varied significantly among the groups, with poorly differentiated tumors exhibiting larger sizes than their well-differentiated counterparts (*p* = 0.010). The number of positive lymph nodes retrieved was highest in poorly differentiated tumors, intermediate in moderately differentiated, and lowest in well-differentiated cases (*p* < 0.001). Accordingly, the lymph node ratio displayed a parallel pattern, with values of 36%, 15%, and 0.5% across these groups (*p* < 0.001).

Membranous/cytoplasmic CD155 expression scores were significantly elevated in poorly differentiated tumors compared with moderately and well-differentiated tumors (*p* = 0.014). In contrast, membranous B7-H3 expression did not differ significantly among the groups; however, higher mean values were consistently observed in poorly differentiated tumors, suggesting a non-significant upward trend. Stromal B7-H3 expression scores also showed no significant differences among groups.

Poorly differentiated tumors were strongly correlated with poorly cohesive histology, consistent with both Lauren and WHO classifications (*p* < 0.001). PNI and LVI occurred more frequently in poorly differentiated tumors, with highly significant intergroup differences (*p* < 0.001 for both). Advanced disease stages, including higher T, N, and overall TNM classifications, were predominantly observed in poorly differentiated tumors (all *p* < 0.001). Metastatic disease was likewise more frequent in this group (*p* = 0.002). Interestingly, ERBB2 positivity occurred more commonly in moderately differentiated tumors (*p* < 0.001). Survival analysis indicated that poorly differentiated tumors were associated with higher mortality rates compared with other groups (*p* = 0.011). Comprehensive data are presented in Table 2 and Supplementary Table S2. A post hoc power analysis using the F test (one-way ANOVA, two-tailed, effect size = 0.4, α = 0.05) demonstrated a statistical power of 0.970, confirming that the sample size of 112 patients was sufficient for reliable inference.

### 3.3. Comparison of Gastric Cancer Patients Based on Pathological Tumor Stage

Tumor diameter averaged 60 mm in advanced-stage tumors and 40 mm in early-stage tumors (*p* < 0.001). The number of retrieved lymph nodes was higher in advanced-stage cases, with a median of 31 compared to 23 in early-stage disease (*p* = 0.024). Similarly, both the number of positive lymph nodes and the lymph node ratio were markedly greater in advanced-stage tumors (*p* < 0.001 for both). Tumor volume also tended to be larger in advanced-stage disease (*p* = 0.011).

Regarding immunohistochemical staining, membranous B7-H3 expression scores were significantly higher in advanced-stage tumors, with a median value of 35 compared to 3 in early-stage disease (*p* = 0.028). Median stromal B7-H3 expression score also exhibited a pronounced increase in advanced-stage tumors, with a median value of 115 vs. 50 in early-stage disease (*p* = 0.001). Likewise, membranous/cytoplasmic CD155 expression score was substantially higher in advanced-stage tumors, median 150 compared to 80 in early-stage disease (*p* < 0.001).

PNI and LVI were almost universally observed in advanced-stage tumors but were rare or absent in early-stage disease (both *p* < 0.001). Advanced-stage tumors were also closely associated with higher pathological N classification, as well as with the presence of metastatic disease (*p* = 0.007). Consequently, most advanced-stage cases were classified as stage III–IV according to the TNM system, whereas early-stage tumors were predominantly staged as I–II (*p* < 0.001). Tumor differentiation patterns varied significantly, with poorly differentiated histology more frequently observed in advanced disease (*p* < 0.001). Patient outcomes were poorer in advanced-stage tumors, as reflected by significantly higher mortality rates compared to early-stage cases (*p* = 0.023). Comprehensive data are presented in Table 3 and Supplementary Table S3. A post hoc power analysis using the *t* test (two-tailed, effect size = 0.8, α = 0.05) demonstrated a statistical power of 0.931, confirming that the number of patients in each group (n = 88 vs. n = 24) was sufficient for reliable inference.

### 3.4. Comparison of Gastric Cancer Patients Based on Tumor Location

Tumor volume was largest in corpus tumors, with a median value of 126 mm^3^, followed by cardia tumors at 63 mm^3^ and distal tumors at 45 mm^3^ (*p* = 0.023). Patients with distal tumors had longer overall follow-up and disease-free follow-up compared with those who had cardia or corpus tumors (*p* = 0.025 and *p* = 0.018, respectively).

Immunohistochemical analysis showed no statistically significant differences in membranous B7-H3, stromal B7-H3, or membranous/cytoplasmic CD155 expression among tumor locations (all *p* > 0.05). Nonetheless, expression levels of these biomarkers tended to be numerically higher in cardia tumors than in corpus or distal tumors, indicating a consistent but non-significant upward trend.

Categorical analyses identified a significant variation in gender distribution (*p* = 0.037), with female patients more frequently presenting with corpus tumors. The type of surgical resection also differed significantly (*p* < 0.001): total gastrectomy was more commonly performed for cardia tumors, whereas subtotal resection predominated in distal tumors. Other categorical comparisons, including Lauren and WHO classification, PNI and LVI, and TNM staging, showed no statistically significant variation across tumor locations. Comprehensive data are summarized in Table 4 and Supplementary Table S4.

### 3.5. Comparison of Gastric Cancer Patients Based on Metastasis Status

Patients with metastatis exhibited a greater number of positive lymph nodes, with a median of 12 compared to 3 in non-metastatic patients (*p* = 0.001). The lymph node ratio was also significantly higher in the metastatic group (31% vs. 17%, *p* = 0.007).

Immunohistochemical analysis demonstrated that all assessed biomarkers were expressed at higher levels in metastatic cases. The median membranous B7-H3 expression score was 60 vs. 20 in non-metastatic tumors (*p* = 0.034). Similarly, stromal B7-H3 expression reached a median of 130 in metastatic tumors vs. 90 in non-metastatic ones (*p* = 0.041). Membranous/cytoplasmic CD155 expression showed the most pronounced difference, with a median of 190 in metastatic vs. 120 in non-metastatic tumors (*p* < 0.001). These findings collectively indicate a strong association between elevated B7-H3 and CD155 expression and the presence of metastasis.

Follow-up analysis revealed shorter overall survival and disease-free survival among patients with metastatic disease. The median overall survival was 498 days compared with 667 days in non-metastatic patients (*p* = 0.049), while the median disease-free survival was 384 vs. 667 days (*p* < 0.001). Categorical comparisons further demonstrated that PNI (90.9% vs. 58.2%, *p* = 0.001) and advanced-stage disease (97.0% vs. 70.9%, *p* = 0.005) were more common in the metastatic group. Higher T stage (*p* = 0.003), and advanced nodal status (N3b, *p* = 0.013) were also significantly associated with metastasis. Patient outcomes were considerably worse, as mortality occurred in 87.9% of metastatic patients compared to 36.7% of those without metastasis (*p* < 0.001). Detailed data are presented in Table 5 and Supplementary Table S5. A post hoc power analysis using the *t* test (two-tailed, effect size = 0.8, α = 0.05) demonstrated a statistical power of 0.968, confirming that the number of patients in each group (n = 33 vs. n = 79) was sufficient for reliable inference.

### 3.6. Comparison of Gastric Cancer Patients Based on Mortality Status

Non-survivors were typically older at the time of diagnosis, with median ages of 68 years compared with 65 years among survivors (*p* = 0.022). Non-survivors also showed a higher burden of nodal disease, having a median of nine positive lymph nodes compared with three in survivors (*p* = 0.001), which corresponded to a greater lymph node ratio of 32% vs. 14% (*p* = 0.001). Tumor volume was markedly greater among non-survivors, measuring 104 mm^3^ compared with 40 mm^3^ in survivors (*p* < 0.001).

Immunohistochemical analysis revealed no statistically significant differences in membranous or stromal B7-H3 and membranous/cytoplasmic CD155 expression scores between survivors and non-survivors (all *p* > 0.05). However, the median scores for membranous B7-H3 (30 vs. 20), stromal B7-H3 (100 vs. 90), and CD155 (145 vs. 130) were consistently higher among non-survivors, indicating a non-significant but biologically plausible upward trend.

Follow-up analyses demonstrated substantial survival disparities. Non-survivors had shorter overall survival, surviving a median of 413 days compared with 911 days in survivors (*p* < 0.001), and shorter disease-free survival, with 378 days compared with 846 days (*p* < 0.001). Categorical analyses revealed that PNI was more frequent among non-survivors, occurring in 82.8% compared with 51.9% of survivors (*p* = 0.001). Advanced disease was also more prevalent in non-survivors, who showed higher rates of stage III–IV classification (87.9% vs. 68.5%, *p* = 0.023), pathological T4b tumors (*p* = 0.040), nodal stage N3b (*p* = 0.007), and distant metastasis (36.2% vs. 7.4%, *p* = 0.001). Consequently, stage IV disease was considerably more common among non-survivors (36.2% vs. 7.4%, *p* < 0.001). Poorly differentiated tumors were also more frequent in non-survivors (67.2% vs. 38.9%, *p* = 0.011). Additionally, recurrence or metastasis was observed far more often in non-survivors (50.0% vs. 7.4%, *p* < 0.001). Detailed findings are presented in Table 6 and Supplementary Table S6. A post hoc power analysis using the *t* test (two-tailed, effect size = 0.8, α = 0.05) demonstrated a statistical power of 0.987, confirming that the number of patients in each group (n = 58 vs. n = 54) was sufficient for reliable inference.

### 3.7. Correlations and Associations of Immunohistochemical Staining Patterns with Invasion Parameters

Correlation analysis revealed significant positive associations among the three immunohistochemical markers. Membranous B7-H3 expression demonstrated a moderate correlation with stromal B7-H3 (r = 0.324, *p* < 0.001) and with membranous/cytoplasmic CD155 (r = 0.281, *p* = 0.003). Likewise, stromal B7-H3 was positively correlated with membranous/cytoplasmic CD155 (r = 0.384, *p* < 0.001), indicating that increased expression of one marker was generally accompanied by higher expression of the others.

Evaluation of invasion parameters showed that all three markers were significantly elevated in tumors exhibiting the presence of PNI or LVI. Membranous B7-H3 expression was higher in tumors with the presence of PNI (*p* = 0.011) and the presence of LVI (*p* = 0.026). Similarly, stromal B7-H3 expression was increased in tumors with the presence of PNI (*p* = 0.003) and the presence of LVI (*p* = 0.001). Membranous/cytoplasmic CD155 expression was also significantly higher in tumors demonstrating the presence of PNI (*p* = 0.001) and the presence of LVI (*p* = 0.005). These findings underscore the strong association between invasive tumor behavior and the increased expression of B7-H3 and CD155. Detailed data are provided in Table 7 and Table 8. A post hoc power analysis using the correlation *t* test (two-tailed, effect size = 0.3, α = 0.05) demonstrated a statistical power of 0.909, confirming that the sample size of 112 patients was sufficient to detect a significant correlation between the two continuous variables.

### 3.8. Gene Expression Analysis Results

Primers were obtained from the Qiagen GeneGlobe database, ensuring standardized assay specificity and reproducibility. A fold change threshold of ≥2 was defined as biologically meaningful upregulation, and a threshold of ≤0.5 as biologically meaningful downregulation. Therefore, interpretations were based primarily on fold change values rather than conventional *p*-values.

Both B7-H3 and CD155 exhibited heterogeneous expression profiles, with B7-H3 demonstrating consistently higher fold change values. B7-H3 expression was upregulated in 75.9% of cases, while CD155 was upregulated in 58.6%. Seventeen patients demonstrated concurrent upregulation of both genes. However, a subset of cases exhibited discordant expression patterns, with CD155 more frequently showing downregulation or divergent expression trends, suggesting that these immune checkpoint molecules may be regulated through distinct or partially independent molecular pathways (Table 9).

**Figure 1:**
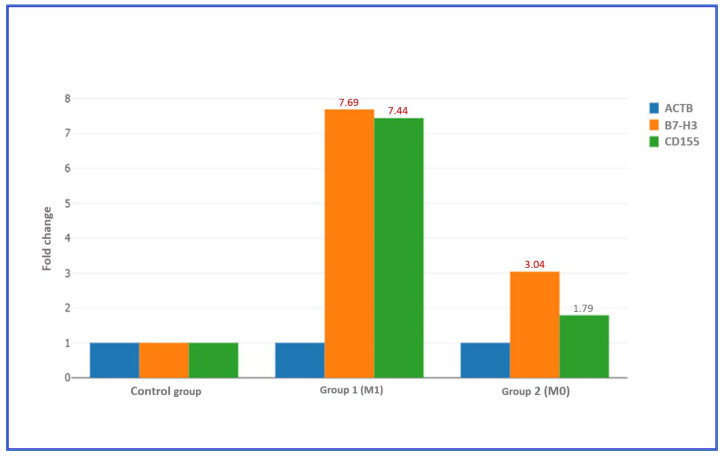
Fold change expression levels of B7-H3 and CD155 across individual gastric cancer cases. A fold change greater than 1 was interpreted as positive or upregulated gene expression, where fold regulation was considered equivalent to the fold change value. A fold change greater than 2 was classified as moderate to high upregulation in gene expression.

**Figure 2:**
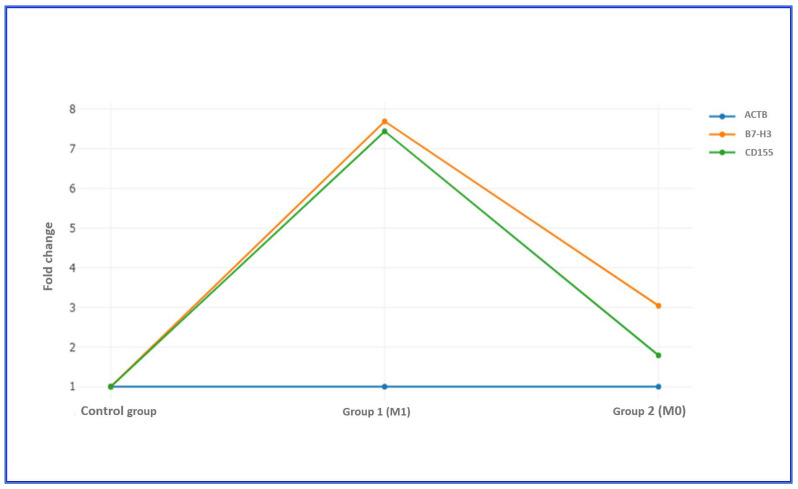
Comparison of B7-H3 and CD155 fold-Change Expression According to Metastatic Status (M0 vs. M1). Fold change was calculated by dividing the normalized gene expression in the test sample by the normalized gene expression in the control sample. Fold regulation represents the biologically relevant direction and magnitude of change derived from the fold change value.

Stratification based on metastatic status revealed that patients with metastasis (M1) exhibited marked overexpression of both genes. B7-H3 showed a 7.69-fold increase and CD155 a 7.44-fold increase, both exceeding the biological significance threshold. In contrast, non-metastatic (M0) patients displayed a 3.04-fold upregulation of B7-H3 and a 1.79-fold increase in CD155. The relative difference between metastatic and non-metastatic groups was greater for CD155 (approximately 4.2-fold) than for B7-H3 (approximately 2.5-fold), indicating that CD155 expression is more strongly associated with the metastatic phenotype (Table 9 and Table 10, Figure 1 and Figure 2).

Based on the differences between B7-H3 and CD155 gene expression levels and the reference housekeeping gene ACTB, metastatic and non-metastatic groups were compared. In both groups, B7-H3 expression levels were markedly upregulated compared with normal controls (*p* < 0.001). The magnitude of B7-H3 overexpression was significantly higher in the M1 group, indicating a strong association between increased gene expression and metastatic progression. A similar expression pattern was observed for CD155, which also demonstrated significantly elevated expression in both groups compared with controls (*p* < 0.001), with a more pronounced fold increase in metastatic cases.

### 3.9. Artificial Intelligence-Based Predictive Modeling of Metastasis and Mortality

The XGBoost-based models developed to predict metastasis and mortality in gastric cancer patients demonstrated strong predictive capability. For metastasis prediction, the model achieved an accuracy of 0.911, a sensitivity of 0.667, and a perfect specificity of 1.000. The positive and negative predictive values were 1.000 and 0.892, respectively, with an F1-score of 0.800. These metrics indicate high reliability in identifying non-metastatic cases while maintaining acceptable sensitivity for metastatic disease (Table 11).

**Figure 3:**
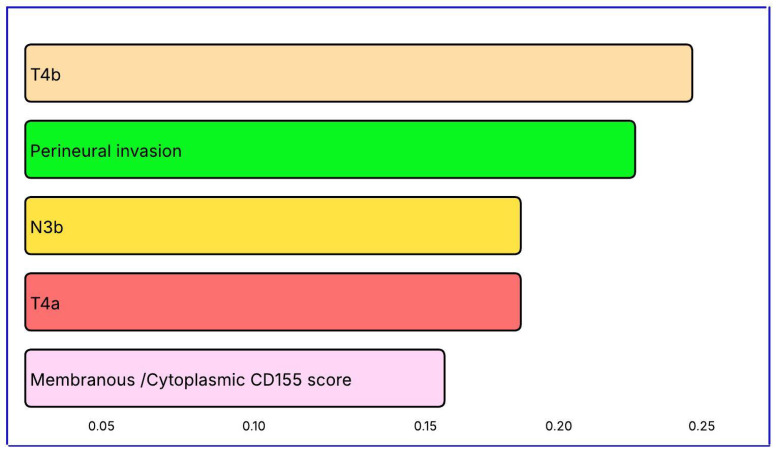
Visualization of variable importance levels in predicting metastasis status with the XGBoost model.

The most influential variables in metastasis prediction were pathological stage T4b (importance score = 0.2409), PNI (0.2217), nodal status N3b (0.1949), stage T4a (0.1889), and membranous/cytoplasmic CD155 expression (0.1616). The contribution of CD155 underscores its biological relevance in metastatic progression (Table 12, Figure 3).

For mortality prediction, the model achieved an accuracy of 0.867, with a sensitivity of 0.826 and specificity of 0.909. The positive predictive value was 0.905 and the negative predictive value 0.833, resulting in an F1-score of 0.864. These findings confirm the model’s robustness in predicting survival outcomes (Table 13).

**Figure 4:**
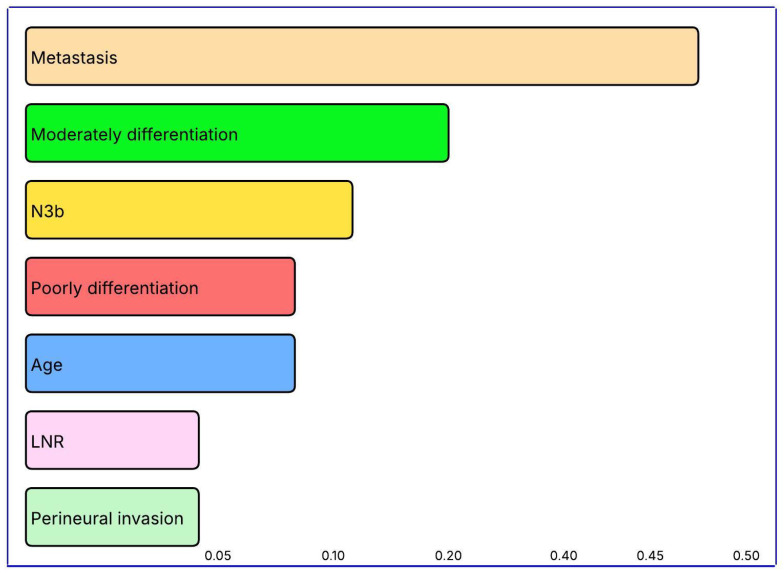
Visualization of variable importance levels in predicting mortality status with the XGBoost model.

The leading predictors of mortality were the presence of metastasis (importance score = 0.4002), moderate differentiation (0.1915), nodal status N3b (0.0989), poor differentiation (0.0949), age (0.0934), lymph node ratio (0.0635), and PNI (0.0575). These results highlight the combined influence of tumor characteristics (differentiation, nodal status, PNI) and patient factors (age) on survival prediction (Table 14, Figure 4).

## 4. Discussion

Molecular biomarkers are increasingly important in gastric cancer for diagnosis, risk assessment, and treatment decisions. ERBB2 overexpression (7–34%) has predictive value, as positivity allows trastuzumab therapy [48]. The prognostic role of PD-L1 remains uncertain, but PD-1 inhibitors may be used in cases with high CPS score [49]. In the present study, median stromal B7-H3 expression was 100 and membranous expression 30. Stromal expression was about 3.3 times higher, indicating its dominant role in the tumor microenvironment. This difference reflects distinct biological functions of B7-H3 in stromal and membranous compartments, which is why both are routinely evaluated in gastric cancer. Our findings, consistent with previous reports, support the dual role of B7-H3 in tumor progression.

Sun et al. [50] showed that membranous B7-H3 expression in tumor cells is linked to adhesion, invasion, and anti-apoptotic mechanisms. Similarly, Zhan et al. [51] found elevated stromal B7-H3 expression in cancer-associated fibroblasts (CAFs), associated with increased angiogenesis and immune evasion. The higher stromal expression observed in our cohort supports the contribution of CAFs to B7-H3 production. Both membranous and stromal B7-H3 expression correlated with several clinicopathological variables, particularly tumor stage and metastasis. Gastric cancers with deeper invasion had higher membranous and stromal B7-H3 scores than early-stage tumors. These results align with Li et al. [20], who reported progressive increases in B7-H3 expression with invasion depth in 120 gastric cancer cases. In their experimental study, silencing B7-H3 in N87 cells did not affect proliferation but reduced migration by about 72% and invasion by 60%. B7-H3 directly interacts with CXCR4 to activate AKT, ERK, and JAK2–STAT3 signaling, enhancing tumor cell motility and invasiveness. These findings indicate that B7-H3 contributes to immune suppression and drives tumor aggressiveness. The pronounced increases observed in T3–T4 cases highlight that elevated B7-H3 promotes motility and invasion independent of proliferation.

Our findings on stromal expression are consistent with Zhan et al. [51], who showed that stromal B7-H3 overexpression in CAFs was linked to poor outcomes. This suggests that elevated stromal B7-H3 remodels the tumor microenvironment to promote invasion. In our cohort, stromal and membranous B7-H3 levels increased together, especially in advanced stages, indicating that deep invasion is not driven solely by intracellular tumor signaling. As invasion depth increased, B7-H3 expression rose in both tumor cells and stroma, supporting its role in tumor progression. Although CXCR4 activation and α-SMA–positive CAFs were not directly measured, the alignment of our data with prior studies supports a consistent pathophysiological mechanism. Overall, higher B7-H3 expression in deeper invasion may serve as a useful biomarker for tumor risk stratification and a potential therapeutic target.

The present study demonstrated that B7-H3 expression is significantly associated with metastasis. Both membranous and stromal B7-H3 scores were higher in metastatic than in non-metastatic patients. Membranous expression increased nearly threefold, while stromal expression rose about 45% in metastatic cases. This pattern suggests that tumor cells and the microenvironment both upregulate B7-H3 in distant metastasis. Rasic et al. [52] proposed that B7-H3 overexpression in gastrointestinal tumors promotes epithelial–mesenchymal transition and upregulates MMP-2/9 and VEGFA, facilitating invasion and angiogenesis. Suppression of B7-H3 experimentally reduces distant metastasis, including pulmonary spread, in colorectal and gastric cancer models. Wang et al. [53] showed that high B7-H3 expression in osteosarcoma was associated with lung metastasis, with 75.8% of metastatic cases showing positivity. Their in vitro work confirmed that B7-H3 enhances invasion through MMP-2 upregulation. Li et al. [20] found that in gastric cancer, B7-H3 interacts with CXCR4 to activate AKT, ERK, and JAK2–STAT3 pathways, enhancing pulmonary metastasis. Silencing B7-H3 suppressed CXCR4 and reduced metastasis. These findings indicate that elevated B7-H3 expression is linked to aggressive disease with distant organ metastasis and may serve as a biomarker of poor prognosis. Parallel increases in membranous and stromal B7-H3 suggest that it reflects reprogramming within the tumor microenvironment that enhances metastatic potential. Predominantly stromal B7-H3 overexpression may serve as a practical high-risk indicator in clinical practice, warranting closer surveillance and earlier systemic therapy.

The present study showed that local invasion features such as LVI and PNI were significantly associated with B7-H3 expression. In patients with LVI, both membranous and stromal B7-H3 expression were higher than in those without. Similarly, tumors with PNI showed significantly higher B7-H3 levels. These results indicate that high B7-H3 expression is linked to local invasion and tumor spread. Stromal B7-H3 upregulation showed a stronger correlation with invasiveness than membranous expression, suggesting stromal cells may produce B7-H3 and promote tumor progression along vascular and neural structures. Although few studies directly examine B7-H3 with PNI or LVI, indirect evidence exists. Zhan et al. [51] reported that B7-H3 overexpression in fibroblasts increased IL-6 and VEGF production, enhancing invasiveness. These cytokines promote angiogenesis and invasion and may contribute to LVI and PNI. The association of stromal B7-H3 with LVI and PNI in this cohort is consistent with these findings. PNI and LVI reflect the tumor’s ability to overcome structural barriers through basement membrane degradation, extracellular matrix remodeling, directional spread, and immune evasion. Ulase et al. [21] showed that such a microenvironment limits CD8+ T-cell activity, facilitating immune escape and tumor spread. In conclusion, coexistence of PNI and LVI with high B7-H3 expression may mark aggressive tumor biology and should be considered when evaluating prognosis. B7-H3-targeted therapy could be integrated into personalized treatment, especially for high-risk patients.

The present study showed that B7-H3 expression was not significantly associated with tumor differentiation. Membranous and stromal B7-H3 scores were similar among poorly, moderately, and well-differentiated adenocarcinomas, with no statistically significant differences. Li et al. [20] also reported no link between B7-H3 and tumor differentiation in 120 patients. Ulase et al. [21] found similar results in 96 cases. In contrast, Chen et al. [36] observed a weak but significant association, noting higher B7-H3 positivity in poorly differentiated tumors. Unlike our study, they grouped well- and moderately differentiated tumors together, and their smaller cohort may explain the discrepancy. The independence of B7-H3 expression from differentiation suggests that its regulation is more closely tied to genetic or immune mechanisms than to histological grade. B7-H3 expression also showed no significant correlation with histopathological subtypes such as intestinal or diffuse types, supporting the idea that its elevation is related to tumor stage and invasion rather than histological category. Similarly, no significant differences in membranous or stromal B7-H3 scores were found among tumors of the cardia, corpus, and distal stomach, indicating that B7-H3 expression is independent of anatomical location. Regarding survival, B7-H3 expression did not have a significant effect. At the end of follow-up, membranous and stromal B7-H3 scores were comparable between survivors and non-survivors.

The present study found that high B7-H3 expression was strongly correlated with advanced stage and metastatic disease. These findings indicate that major prognostic factors—stage, metastasis, and PNI—are associated with B7-H3 expression. Since B7-H3 was not directly related to overall survival, it may not function as an independent prognostic marker but instead influence outcomes indirectly through its correlation with established factors. Ulase et al. [21] also found no independent association between B7-H3 and overall survival. In contrast, larger cohort studies reported shorter survival in patients with high B7-H3 expression. Chen et al. [36], for example, demonstrated significantly reduced overall survival in such patients. The absence of statistical significance in our analysis may reflect the limited sample size and the dominant prognostic impact of disease stage. Although results in the literature are mixed, most studies link high B7-H3 expression to poor prognosis. Our findings indirectly support this association. To clarify the independent prognostic role of B7-H3, larger multicenter studies with long-term follow-up are warranted.

CD155, a type I transmembrane glycoprotein of the nectin-like family, is an important immune checkpoint molecule in gastric cancer [54]. Our study found that CD155 expression was significantly associated with several clinicopathological parameters but showed no correlation with some traditional prognostic factors, suggesting a more complex role than previously reported. CD155 displayed consistently strong membranous expression across gastric cancer samples. In contrast, membranous B7-H3 scores varied widely, with some tumors showing low and others very high expression. This variability implies that B7-H3 may help identify biological subgroups, while CD155 serves as a more stable marker across the cohort. Liu et al. [54] reported a progressive increase in CD155 expression from gastritis to high-grade intraepithelial neoplasia, suggesting its role in gastric carcinogenesis. In gastric cancer tissues, CD155 was markedly overexpressed, and TCGA-STAD analysis confirmed significantly higher mRNA levels in tumors compared to normal controls. CD155 overexpression correlated positively with tumor volume, stage, and lymph node involvement, and was significantly associated with poor overall survival. Overall, these findings indicate that high CD155 expression in gastric cancer is linked to aggressive behavior and poor prognosis, supporting its potential as a clinically relevant biomarker.

One of the most notable findings of this study was the strong correlation between membranous CD155 score and tumor differentiation. Median CD155 levels were 150 in poorly differentiated tumors, 130 in moderately differentiated tumors, and 95 in well-differentiated tumors, showing a clear increase as differentiation decreased. This suggests that CD155 is linked to tumor aggressiveness and dedifferentiation. In a study of 268 gastric cancer patients, Liu et al. [54] reported a similar pattern, with CD155 expression rising from precancerous lesions to invasive carcinoma and correlating with dedifferentiation. Their TCGA-STAD analysis also showed that CD155 expression was significantly associated with tumor stage and differentiation. In our cohort, the gradual increase in membranous/cytoplasmic CD155 with poorer differentiation was consistent with higher LN ratio, advanced TNM stage (T4b/N3b/M1), and shorter survival in the poorly differentiated group.

This study revealed significantly higher membranous and cytoplasmic CD155 expression in metastatic patients, underscoring CD155’s role as a regulator of metastatic progression. Liu et al. [54] reported that CD155 expression correlates with tumor volume, stage, and lymph node involvement, and that elevated CD155 is linked to poorer overall survival. CD155 has also been implicated in peritoneal metastasis. Ng et al. [55] identified CD155 as a key molecule in gastric peritoneal metastasis, facilitating tumor cell adhesion to peritoneal mesothelial cells. This suggests that CD155’s clinical importance extends beyond immune checkpoint regulation to direct involvement in metastatic colonization. Similarly, Bai et al. [56] showed in gastric cancer models that CD155 promotes cell migration, invasion, and fibronectin binding, supporting its role in peritoneal dissemination. Our finding of increased CD155 expression in metastatic cases aligns with these reports and reinforces its potential as a biomarker for metastatic risk and a therapeutic target. Another notable observation was the strong correlation between CD155 expression and tumor invasion depth. In advanced-stage tumors, the CD155 H-score averaged 150 compared with 80 in early-stage tumors, reflecting an 87% increase. This highlights the contribution of CD155 to gastric wall infiltration and invasive potential. Similar associations between high CD155 expression and advanced disease have been observed in melanoma, glioblastoma, colorectal, pancreatic, and lung cancers [25]. Overall, elevated CD155 expression in advanced and metastatic gastric cancer emphasizes its importance as a marker of aggressiveness and a promising therapeutic target.

The XGBoost-based metastasis classifier showed strong predictive performance, achieving 91.1% accuracy and an F1-score of 0.80. Both specificity and positive predictive value reached 100%, indicating no false-positive predictions for metastasis. This suggests the model can be reliably applied in clinical settings that require confirmation of metastatic disease, where high specificity is critical. The top five predictors were pathological stage T4b, PNI, nodal status N3b, pathological stage T4a, and membranous/cytoplasmic CD155 score. As expected, advanced T stage (T4a–T4b) and high nodal burden (N3b) were the strongest pathological indicators of metastasis. The high ranking of PNI emphasizes its role in local invasion and metastatic spread. The inclusion of the membranous/cytoplasmic CD155 score among the top variables underscores the biological and clinical relevance of this immune checkpoint in metastasis. Overall, integrating pathological parameters with immune biomarkers enhances prognostic accuracy and provides improved risk stratification for metastatic gastric cancer.

LVI and PNI are among the most important prognostic indicators in gastric cancer. This study is, to our knowledge, the first to directly examine the relationship between B7-H3 and CD155 expression and these invasive features in gastric adenocarcinoma. A clear association was observed for both markers, suggesting that increased expression accompanies enhanced invasive capacity. Cases with LVI or PNI consistently showed higher CD155 levels, emphasizing its role in promoting angiogenesis and neural invasion. Few previous studies have directly assessed this relationship, but our results demonstrate that elevated CD155 expression parallels increased invasiveness. The association of high CD155 levels with these features is consistent with prior reports describing an aggressive phenotype. Previous research has also linked CD155 overexpression to advanced stage, lymph node metastasis, and angiogenesis [54]. Collectively, these findings indicate that CD155 supports tumor cell migration and contributes to a microenvironment that facilitates spread through vascular, lymphatic, and neural pathways.

Previous studies on gastrointestinal cancers [57,58,59], including that of Liu et al. [54], have shown a strong relationship between high CD155 expression and features such as larger tumor size, advanced stage, and lymph node metastasis. Our findings demonstrate a similar pattern in gastric adenocarcinoma, indicating that increased CD155 expression accompanies more aggressive tumor behavior. The observation of elevated CD155 levels in tumors exhibiting LVI and PNI further supports its role in promoting both metastatic spread and local infiltration within the tumor microenvironment.

The RT-PCR fold-change data from this study provide an overview of B7-H3 (CD276) and CD155 (PVR) gene expression in gastric cancer. Quantitative RT-PCR analysis of 29 gastric adenocarcinoma cases showed upregulation of both genes and a strong correlation between them. These findings align with the existing literature and highlight their synergistic role in gastric cancer pathogenesis. A fold-change threshold of 2, an accepted standard in RT-PCR analyses, was used. The comparative Ct method (Livak and Schmittgen) [47] remains the gold standard for measuring gene expression. High sensitivity, reproducibility, and rapid turnaround make RT-PCR an ideal tool for validating gene expression and translating results into clinical use. In the era of immunotherapy, quantitative measurement of checkpoint molecule expression is essential for treatment selection and response monitoring. This method supports personalized therapy and companion diagnostics. RT-PCR can detect a wide range of expression changes, distinguishing even minimal differences between samples. Both B7-H3 and CD155 showed expression levels above the threshold, confirming their upregulation and clinical relevance for risk assessment and prognosis [60]. Concurrent overexpression of both B7-H3 and CD155 in more than half of the cases suggests that combined evaluation offers greater prognostic value than assessing each marker alone. Overall, RT-PCR analysis of B7-H3 and CD155 expression provides insight into gastric cancer biology and demonstrates clinical potential.

Immune-regulatory genes such as B7-H3 and CD155 show notable differences between tumor and normal tissues. In this study, both genes were expressed at much higher levels in tumor tissues than in normal gastric samples. Previous studies evaluating mRNA levels by qRT-PCR consistently reported that high expression is linked to disease progression and poor prognosis [54,61]. Sun et al. [62] examined B7-H3 expression in gastric cancer using mRNA extracted from tumor and adjacent non-tumor tissues. Their analysis of 80 samples revealed marked upregulation of B7-H3 in tumors, with a clear association between higher expression and advanced clinical stage. Liu et al. [54] used TCGA data and found significantly elevated CD155 expression in gastric adenocarcinoma compared with normal tissue, which was also correlated with poor overall survival. Together, these findings suggest that CD155 expression has clinical relevance as a biomarker of disease progression and as a potential therapeutic target in gastric cancer.

qRT-PCR analyses of tissue and blood samples in gastric cancer have consistently shown elevated B7-H3 mRNA levels, correlating with advanced stage, metastasis, and reduced survival [61,62]. Similarly, increased CD155 mRNA expression has been observed in various cancers, including gastric cancer, where it correlates with tumor stage, lymph node metastasis, and poor survival [57]. The upregulation of B7-H3 and CD155 identified by RT-PCR provides insight into tumor biology and prognosis. The literature supports their role as prognostic biomarkers based on qRT-PCR findings [54,61]. In the present study, B7-H3 upregulation was more pronounced than CD155, suggesting stronger involvement in tumor progression. Overexpression of both molecules likely enhances immune suppression and therapy resistance. Thus, B7-H3 and CD155 represent not only prognostic indicators but also potential therapeutic targets. In patients resistant to PD-1/PD-L1 blockade, these molecules may serve as alternative immunotherapy targets [62,63].

Distant organ metastasis is a major prognostic factor in gastric cancer. In this study, the strong association between B7-H3 and CD155 expression and metastasis observed in immunohistochemistry was confirmed at the mRNA level using RT-PCR. Both genes showed higher expression in metastatic patients compared with non-metastatic ones. B7-H3 and CD155 expression levels in the metastatic group exceeded the accepted biological significance threshold, supporting their role in aggressive disease behavior. Although based on a limited number of cases, this study is the first to assess B7-H3 and CD155 mRNA levels together by RT-PCR in gastric cancer. Our findings show a clear relationship between the co-expression of CD155 and B7-H3 and metastasis confirmed by RT-PCR, highlighting their value in prognostic risk assessment.

The relationship between B7-H3 and CD155 expression in gastric cancer was further examined to explore potential co-regulation and interaction mechanisms. Membranous and stromal B7-H3 scores and membranous/cytoplasmic CD155 scores obtained by immunohistochemistry were analyzed using correlation analysis. A significant positive correlation was found between membranous and stromal B7-H3, as well as between membranous B7-H3 and CD155 expression, suggesting coordinated upregulation of these molecules within the tumor microenvironment. To the best of our knowledge, this association has not been previously investigated in gastric or other gastrointestinal cancers. The findings imply that B7-H3 and CD155 may act synergistically to promote tumor progression through shared signaling pathways and microenvironmental interactions.

### Limitations

This study has several limitations. The modest sample size, especially in subgroup analyses, may have reduced the power to detect small effects. Its retrospective, single-center design also introduces potential selection bias and limits generalizability. Although RT-PCR analysis provided molecular validation of immunohistochemical results, functional assays were not performed to clarify the biological mechanisms involved. The absence of external validation further restricts the robustness of the conclusions. Due to economic constraints, RT-PCR could not be performed for all patients, which represents an additional limitation. Differences in tissue fixation and staining protocols may have introduced technical variability that could influence staining intensity and scoring consistency. Moreover, the study did not assess tumor microenvironment components such as immune cell infiltration, limiting interpretation of the biological interactions underlying B7-H3 and CD155 expression. Future large-scale, multicenter studies with integrated functional and immune microenvironment analyses are warranted to validate and extend these findings.

## 5. Conclusions

This study provides robust evidence that B7-H3 and CD155 are co-upregulated in gastric adenocarcinoma, as demonstrated by both immunohistochemical and quantitative RT-PCR analyses. Elevated expression of these immune checkpoint molecules was significantly associated with advanced pathological stage, poor differentiation, the presence of lymphovascular and perineural invasion, and metastatic disease. The strong concordance between protein and mRNA expression supports their role as biologically meaningful markers of tumor aggressiveness. The findings collectively indicate that B7-H3 and CD155 act synergistically to establish an immunosuppressive tumor microenvironment that facilitates invasion and metastasis. Their combined assessment enhances prognostic stratification beyond conventional pathological parameters and may serve as a foundation for precision immunotherapeutic approaches. Targeting the B7-H3 pathway together with the CD155 signaling axis may represent a promising concurrent blockade strategy for improving survival outcomes in patients with gastric adenocarcinoma. Future large-scale, multicenter, and functionally validated studies are warranted to further define the therapeutic implications of concurrent B7-H3 and CD155 blockade and to explore their integration into personalized treatment algorithms.

## Figures and Tables

**Table 1:** Descriptive statistics of continuous variables in the overall patient cohort.

Variables [Median (95% CI)]	Results (n = 112)
Age (years)	65 (63–68)
Tumor Diameter (mm)	56 (50–65)
Total LN retrieved (number)	30 (28–33)
Positive LN retrieved (number)	7 (5–10)
LNR	25 (17–32)
Tumor Volume	60 (45–103)
B7H3 score (membranous)	30 (20–60)
B7H3 score (stromal)	100 (90–130)
CD155 score (membranous/cytoplasmic)	140 (130–170)
Overall follow-up (days)	615 (496–771)
Disease-free follow-up (days)	542 (445–690)

LN: Lymph node; LNR: Lymph node ratio.

**Table 2:** Comparison of continuous variables according to tumor differentiation characteristics.

Variables [Median (95% CI)]	Poorly	Moderately	Well	*p*
Age (years)	64 (59–68)	66 (65–70)	67 (59–74)	0.613
Tumor Diameter (mm)	60 (55–70)	55 (45–75)	45 (35–70)	0.010
Total LN retrieved (number)	30 (28–36)	32 (28–38)	24 (20–40)	0.100
Positive LN retrieved (number)	11 (9–13)	4 (3–7)	1 (0–5)	<0.001
LNR	36 (28–43)	15 (11–28)	0.5 (0–17)	<0.001
Tumor Volume	60 (45–126)	86 (41–120)	42 (24–100)	0.194
B7-H3 score (membranous)	43 (20–80)	20 (10–60)	20 (0–60)	0.380
B7-H3 score (stromal)	100 (80–140)	120 (100–140)	60 (40–120)	0.147
CD155 score (membranous/cytoplasmic)	150 (140–190)	130 (100–160)	95 (30–140)	0.014
Overall follow-up (days)	502 (394–835)	943 (708–1303)	610 (436–925)	0.046
Disease-free follow-up (days)	484 (375–728)	728 (631–1204)	564 (384–925)	0.105

LN: Lymph node; LNR: Lymph node ratio.

**Table 3:** Comparison of continuous variables according to pathological tumor stage (pT).

Variables [Median (95% CI)]	Advanced Stage (n = 88)	Early Stage (n = 24)	*p*
Age (years)	66 (63–69)	65 (61–72)	0.502
Tumor Diameter (mm)	60 (55–70)	40 (30–50)	<0.001
Total LN retrieved (number)	31 (29–36)	23 (18–33)	0.024
Positive LN retrieved (number)	9 (7–12)	1 (0–2)	<0.001
LNR	31 (22–38)	1 (0–12)	<0.001
Tumor Volume	76 (50–112)	41 (21–56)	0.011
B7-H3 score (membranous)	35 (20–60)	3 (0–30)	0.028
B7-H3 score (stromal)	115 (100–140)	50 (10–90)	0.001
CD155 score (membranous/cytoplasmic)	150 (140–180)	80 (35–120)	<0.001
Overall follow-up (days)	615 (502–728)	934 (496–1220)	0.263
Disease-free follow-up (days)	542 (468–687)	934 (496–1210)	0.115

LN: Lymph node; LNR: Lymph node ratio.

**Table 4:** Comparison of continuous variables according to tumor location.

Variables [Median (95% CI)]	Cardia	Corpus	Distal	*p*
Age (years)	65 (58–70)	70 (66–75)	65 (62–70)	0.105
Tumor Diameter (mm)	50 (45–65)	65 (55–90)	53 (45–65)	0.052
Total LN retrieved (number)	28 (23–33)	29 (28–38)	30 (27–40)	0.677
Positive LN retrieved (number)	5 (2–11)	8 (2–12)	7 (3–12)	0.756
LNR	19 (9–37)	28 (15–46)	27 (14–32)	0.849
Tumor Volume	63 (36–105)	126 (56–256)	45 (39–97)	0.023
B7-H3 score (membranous)	53 (20–100)	20 (10–90)	30 (10–60)	0.730
B7-H3 score (stromal)	125 (85–150)	100 (40–130)	100 (80–140)	0.277
CD155 score (membranous/cytoplasmic)	155 (95–175)	140 (90–190)	130 (120–150)	0.963
Overall follow-up (days)	496 (303–622)	607 (385–961)	896 (708–1002)	0.025
Disease-free follow-up (days)	433 (294–554)	510 (372–771)	796 (687–975)	0.018

LN: Lymph node; LNR: Lymph node ratio.

**Table 5:** Comparison of continuous variables according to metastasis status.

Variables [Median (95% CI)]	Metastasis (+)	Metastasis (−)	*p*
Age (years)	66 (59–68)	65 (65–70)	0.612
Tumor Diameter (mm)	60 (55–75)	55 (50–65)	0.249
Total LN retrieved (number)	34 (28–45)	29 (28–35)	0.199
Positive LN retrieved (number)	12 (9–23)	3 (2–7)	0.001
LNR	31 (22–55)	17 (11–31)	0.007
Tumor Volume	86 (45–126)	56 (41–100)	0.239
B7-H3 score (membranous)	60 (30–100)	20 (5–60)	0.034
B7-H3 score (stromal)	130 (100–160)	90 (60–110)	0.041
CD155 score (membranous/cytoplasmic)	190 (150–220)	120 (100–140)	<0.001
Overall follow-up (days)	498 (303–849)	667 (527–896)	0.049
Disease-free follow-up (days)	384 (278–523)	667 (527–896)	<0.001

LN: Lymph node; LNR: Lymph node ratio.

**Table 6:** Comparison of continuous variables according to mortality status.

Variables [Median (95% CI)]	Non-Survivor (n = 58)	Survivor (n = 54)	*p*
Age (years)	68 (65–73)	65 (61–70)	0.022
Tumor Diameter (mm)	60 (55–75)	53 (45–65)	0.108
Total LN retrieved (number)	31 (28–40)	29 (24–32)	0.238
Positive LN retrieved (number)	9 (7–13)	3 (1–7)	0.001
LNR	32 (22–43)	14 (6–28)	0.001
Tumor Volume	104 (60–126)	40 (27–56)	<0.001
B7-H3 score (membranous)	30 (20–80)	20 (10–60)	0.415
B7-H3 score (stromal)	100 (90–140)	90 (60–120)	0.114
CD155 score (membranous/cytoplasmic)	145 (120–180)	130 (120–170)	0.066
Overall follow-up (days)	413 (298–530)	911 (667–1220)	<0.001
Disease-free follow-up (days)	378 (278–498)	846 (650–1210)	<0.001

LN: Lymph node; LNR: Lymph node ratio.

**Table 7:** Correlations among immunohistochemical staining scores.

		B7-H3 (Membr)	B7-H3 (Stromal)	CD155 (Membr/Cytopl)
B7-H3 score (membr)	r	1.000	0.324	0.281
	*p*	.	0.000	0.003
B7-H3 score (stromal)	r	0.324	1.000	0.384
	*p*	0.000	.	0.000
CD155 score (membr/cytopl)	rı	0.281	0.384	1.000
	*p*	0.003	0.000	.

Memebr: Membranous; Cytopl: Cytoplasmic.

**Table 8:** Comparison of immunohistochemical staining pattern scores according to the presence of PNI and LVI.

Variables	Status	n	Median	95%CI	*p*
B7-H3 score (membranous)					
PNI	Present	76	43	20–60	0.009
LVI	Present	99	30	20–60	0.023
B7-H3 score (stromal)					
PNI	Present	76	120	100–140	0.002
LVI	Present	99	110	100–130	0.001
CD155 score (membranous/cytoplasmic)					
PNI	Present	76	150	140–180	<0.001
LVI	Present	99	140	130–170	0.003

LVI: Lymphovascular invasion; PNI: Perineural invasion.

**Table 9:** Evaluation of fold change expression levels of B7-H3 and CD155 according to the Livak method.

Group	ACTB	B7-H3	CD155
Position	1.00	2	3
Case 1	1.00	10.55	28.26
Case 2	1.00	7.78	19.92
Case 3	1.00	7.86	26.10
Case 4	1.00	9.38	11.01
Case 5	1.00	13.08	36.15
Case 6	1.00	12.04	15.84
Case 7	1.00	18.75	4.76
Case 8	1.00	8.14	13.32
Case 9	1.00	2.50	0.29
Case 10	1.00	4.21	5.01
Case 11	1.00	4.33	1.08
Case 12	1.00	3.58	0.50
Case 13	1.00	1.03	4.73
Case 14	1.00	1.87	1.31
Case 15	1.00	7.46	4.17
Case 16	1.00	1.02	0.23
Case 17	1.00	0.88	1.98
Case 18	1.00	2.50	0.48
Case 19	1.00	0.24	1.68
Case 20	1.00	7.46	0.70
Case 21	1.00	1.62	0.33
Case 22	1.00	2.57	0.21
Case 23	1.00	4.92	2.41
Case 24	1.00	0.45	0.05
Case 25	1.00	5.63	10.60
Case 26	1.00	9.15	8.82
Case 27	1.00	11.51	13.28
Case 28	1.00	11.83	9.65
Case 29	1.00	31.00	48.36

**Table 10:** Evaluation of fold change expression levels of B7-H3 and CD155 according to metastasis status.

Position	Symbol	M1 Fold Change	M0 Fold Change
1	ACTB	1.0	1.0
2	B7-H3	7.69	3.04
3	CD155	7.44	1.79

**Table 11:** Performance metrics for classification of metastasis status using the XGBoost model.

Metrics	Value
Accuracy	0.911
Sensitivity	0.667
Specificity	1.000
PPV	1.000
NPV	0.892
F1 Score	0.800

PPV: Positive predictive value, NPV: Negative predictive value.

**Table 12:** Most influential variables in predicting metastasis status according to the XGBoost model (importance scores).

Variables	Value
T4b	0.2409
PNI	0.2217
N3b	0.1949
T4a	0.1889
CD155 (membranous/cytoplasmic) score	0.1616

**Table 13:** Performance metrics for classification of mortality status using the XGBoost model.

Metrics	Value
Accuracy	0.867
Sensitivity	0.826
Specificity	0.909
PPV	0.905
NPV	0.833
F1 Score	0.864

PPV: Positive predictive value, NPV: Negative predictive value.

**Table 14:** Most influential variables in predicting mortality status according to the XGBoost model (importance scores).

Variable	Value
M1	0.4002
Moderately differentiation	0.1915
N3b	0.0989
Poorly differentiation	0.0949
Age	0.0934
LNR	0.0635
PNI	0.0575

## Data Availability

Data supporting the findings of this study are available within the article and Supplementary Materials. Additional de-identified data may be provided upon reasonable request to the corresponding author.

## Supplementary Materials

The following supporting information can be downloaded at: https://www.mdpi.com/article/10.3390/diagnostics15212695/s1, Table S1: Distribution of categorical variables in the overall patient cohort; Table S2: Comparison of categorical variables according to tumor differentiation characteristics; Table S3: Comparison of categorical variables according to pathological tumor stage (pT); Table S4: Comparison of categorical variables according to tumor location; Table S5: Comparison of categorical variables according to metastasis status; Table S6: Comparison of categorical variables according to mortality status. Figure S1: CD155 (PVR) expression in normal gastric tissue and gastric carcinoma, CD155 × 200. A: Normal gastric tissue shows no CD155 expression. B: Tubulary gastric carcinoma shows weak and moderate (C) CD155 expression. D–F: strong CD155 expression in signet cell (D), poorly differentiated tubular (E), and papillary carcinoma (F). Figure S2: B7-H3 (CD276) expression in normal gastric tissue and gastric carcinoma, B7-H3 × 200. A: Normal gastric tissue shows no B7-H3 expression. B: Gastric carcinoma shows no expression in epithelial component whereas stromal cells show positive expression. C: Moderate tumoral epithelial expression in tubulary gastric carcinoma. D,E: Strong tumoral epithelial expression in gastric carcinoma. F: Strong epithelial and stromal expression in gastric carcinoma.
