# Supplementary material for: Prognostic and Predictive Significance of B7-H3 and CD155 Expression in Gastric Cancer Patients
Source: Diagnostics (Basel). 2025 Oct 24;15(21):2695. doi: 10.3390/diagnostics15212695 (PMC12607427; doi:10.3390/diagnostics15212695)
Supplement: Supplementary file 1 [file diagnostics-15-02695-s001.zip › diagnostics-3931316-supplementary.pdf]

Table S1: Distribution of categorical variables in the overall patient cohort

**Variables**	**Categories**	**n (%)**
Gender	Female	32 (28.6)
	Male	80 (71.4)
Tumor Location	Cardia	28 (25.0)
	Corpus	29 (25.9)
	Distal	55 (49.1)
Type of Resection	Total	57 (50.9)
	Near Total	5 (4.5)
	Subtotal	50 (44.6)
Lauren Classification	Poorly cohesive	24 (21.4)
	Mixed	21 (18.8)
	Intestinal	67 (59.8)
WHO Classification	Poorly cohesive	24 (21.4)
	Mixed adenoca	21 (18.8)
	Tubular adenoca	54 (48.2)
	Papillary adenoca	11 (9.8)
	Mucinous adenoca	2 (1.8)
Perineural Invasion	Present	76 (67.9)
	Absent	36 (32.1)
Lymphovascular Invasion	Present	99 (88.4)
	Absent	13 (11.6)
Tumor Stage	Advanced stage	88 (78.6)
	Early stage	24 (21.4)
Pathological T Stage	T4b	13 (11.6)
	T4a	39 (34.8)
	T3	36 (32.1)
	T2	12 (10.7)
	T1b	9 (8.0)
	T1a	3 (2.7)
Pathological N Stage	N3b	22 (19.6)
	N3a	34 (30.4)
	N2	14 (12.5)
	N1	24 (21.4)
	N0	18 (16.1)
Clinical M Stage	M1	25 (22.3)
	M0	87 (77.7)

TNM Staging	IV	25 (22.3)
	IIIC	16 (14.3)
	IIIB	17 (15.2)
	IIIA	18 (16.1)
	IIB	11 (9.8)
	IIA	11 (9.8)
	IB	7 (6.3)
	IA	7 (6.3)
Tumor Differentiation	Poorly	60 (53.6)
	Moderately	31 (27.7)
	Well	21 (18.8)
HER2/neu staining	Positive	15 (13.4)
	Negative	97 (86.6)
Metastasis/Recurrence	Present	33 (29.5)
	Absent	79 (70.5)
Outcome	Non-survivors	58 (51.8)
	Survivors	54 (48.2)

Table S2: Comparison of categorical variables according to tumor differentiation characteristics

Variables	Categories	Poorly	Moderately	Well	p
Gender	Female	15 (25.0)	11 (35.5)	6 (28.6)	0.577
	Male	45 (75.0)	20 (64.5)	15 (71.4)	
Tumor Location	Cardia	13 (21.7)	6 (19.4)	9 (42.9)	0.332
	Corpus	17 (28.3)	8 (25.8)	4 (19.0)	
	Distal	30 (50.0)	17 (54.8)	8 (38.1)	
Type of Resection	Total	34 (56.7)	11 (35.5)	12 (57.1)	0.052
	Near Total	1 (1.7)	4 (12.9)	0 (0.0)	
	Subtotal	25 (41.7)	16 (51.6)	9 (42.9)	
Lauren Classification	Poorly cohesive	24 (40.0)	0 (0.0)	0 (0.0)	<0.001
	Mixed	10 (16.7)	9 (29.0)	2 (9.5)	
	Intestinal	26 (43.3)	22 (71.0)	19 (90.5)	
WHO Classification	Poorly cohesive	24 (40.0)	0 (0.0)	0 (0.0)	<0.001
	Mixed adenoca	10 (16.7)	9 (29.0)	2 (9.5)	
	Tubular adenoca	25 (41.7)	15 (48.4)	14 (66.7)	
	Papillary adenoca	1 (1.7)	5 (16.1)	5 (23.8)	
	Mucinous adenoca	0 (0.0)	2 (6.5)	0 (0.0)	
Perineural Invasion	Present	51 (85.0)	18 (58.1)	7 (33.3)	<0.001
	Absent	9 (15.0)	13 (41.9)	14 (66.7)	
Lymphovascular Invasion	Present	59 (98.3)	29 (93.5)	11 (52.4)	<0.001
	Absent	1 (1.7)	2 (6.5)	10 (47.6)	
Tumor Stage	Advanced stage	51 (85.0)	18 (58.1)	7 (33.3)	<0.001
	Early stage	9 (15.0)	13 (41.9)	14 (66.7)	
Pathological T Stage	T4b	12 (20.0)	1 (3.2)	0 (0.0)	<0.001
	T4a	25 (41.7)	11 (35.5)	3 (14.3)	
	T3	17 (28.3)	14 (45.2)	5 (23.8)	
	T2	4 (6.7)	3 (9.7)	5 (23.8)	
	T1b	2 (3.3)	2 (6.5)	5 (23.8)	
	T1a	0 (0.0)	0 (0.0)	3 (14.3)	
Pathological N Stage	N3b	17 (28.3)	4 (12.9)	1 (4.8)	<0.001
	N3a	25 (41.7)	7 (22.6)	2 (9.5)	
	N2	4 (6.7)	9 (29.0)	1 (4.8)	
	N1	7 (11.7)	10 (32.3)	7 (33.3)	
	N0	7 (11.7)	1 (3.2)	10 (47.6)	
Clinical M Stage	M1	21 (35.0)	3 (9.7)	1 (4.8)	0.002

	M0	39 (65.0)	28 (90.3)	20 (95.2)	
TNM Staging	IV	21 (35.0)	3 (9.7)	1 (4.8)	<0.001
	IIIC	8 (13.3)	6 (19.4)	2 (9.5)	
	IIIB	14 (23.3)	3 (9.7)	0 (0.0)	
	IIIA	8 (13.3)	8 (25.8)	2 (9.5)	
	IIB	2 (3.3)	5 (16.1)	4 (19.0)	
	IIA	5 (8.3)	6 (19.4)	0 (0.0)	
	IB	1 (1.7)	0 (0.0)	6 (28.6)	
	IA	1 (1.7)	0 (0.0)	6 (28.6)	
HER2/neu staining	Positive	1 (1.7)	10 (32.3)	4 (19.0)	<0.001
	Negative	59 (98.3)	21 (67.7)	17 (81.0)	
Metastasis/Recurrence	Present	22 (36.7)	8 (25.8)	3 (14.3)	0.134
	Absent	38 (63.3)	23 (74.2)	18 (85.7)	
Outcome	Non-survivors	39 (65.0)	11 (35.5)	8 (38.1)	0.011
	Survivors	21 (35.0)	20 (64.5)	13 (61.9)	

Table S3: Comparison of categorical variables according to pathological tumor stage (pT)

Variables	Categories	Advanced stage	Early stage	p
Gender	Female	23 (26.1)	9 (37.5)	0.402**
	Male	65 (73.9)	15 (62.5)	
Tumor Location	Cardia	21 (23.9)	7 (29.2)	0.771*
	Corpus	24 (27.3)	5 (20.8)	
	Distal	43 (48.9)	12 (50.0)	
Type of Resection	Total	48 (54.5)	9 (37.5)	0.307*
	Near Total	4 (4.5)	1 (4.2)	
	Subtotal	36 (40.9)	14 (58.3)	
Lauren Classification	Poorly cohesive	20 (22.7)	4 (16.7)	0.058*
	Mixed	20 (22.7)	1 (4.2)	
	Intestinal	48 (54.5)	19 (79.2)	
WHO Classification	Poorly cohesive	20 (22.7)	4 (16.7)	0.068*
	Mixed adenoca	20 (22.7)	1 (4.2)	
	Tubular adenoca	40 (45.5)	14 (58.3)	
	Papillary adenoca	6 (6.8)	5 (20.8)	
	Mucinous adenoca	2 (2.3)	0 (0.0)	
Perineural Invasion	Present	76 (86.4)	0 (0.0)	<0.001**
	Absent	12 (13.6)	24 (100.0)	
Lymphovascular Invasion	Present	87 (98.9)	12 (50.0)	<0.001***
	Absent	1 (1.1)	12 (50.0)	
Pathological T Stage	T4b	13 (14.8)	0 (0.0)	<0.001**
	T4a	39 (44.3)	0 (0.0)	
	T3	36 (40.9)	0 (0.0)	
	T2	0 (0.0)	12 (50.0)	
	T1b	0 (0.0)	9 (37.5)	
	T1a	0 (0.0)	3 (12.5)	
Pathological N Stage	N3b	22 (25.0)	0 (0)	<0.001**
	N3a	31 (35.2)	3 (12.5)	
	N2	12 (13.6)	2 (8.3)	
	N1	17 (19.3)	7 (29.2)	
	N0	6 (6.8)	12 (50.0)	
Clinical M Stage	M1	25 (28.4)	0 (0.0)	0.007*
	M0	63 (71.6)	24 (100.0)	
TNM Staging	IV	25 (28.4)	0 (0.0)	<0.001

	IIIC	16 (18.2)	0 (0.0)	
	IIIB	16 (18.2)	1 (4.2)	
	IIIA	16 (18.2)	2 (8.3)	
	IIB	11 (12.5)	0 (0.0)	
	IIA	4 (4.5)	7 (29.2)	
	IB	0 (0.0)	7 (29.2)	
	IA	0 (0.0)	7 (29.2)	
Tumor Differentiation	Poorly	54 (61.4)	6 (25.0)	<0.001
	Moderately	26 (29.5)	5 (20.8)	
	Well	8 (9.1)	13 (54.2)	
HER2/neu staining	Positive	10 (11.4)	5 (20.8)	0.307***
	Negative	78 (88.6)	19 (79.2)	
Metastasis/ Recurrence	Present	32 (36.4)	1 (4.2)	0.005**
	Absent	56 (63.6)	23 (95.8)	
Outcome	Non-survivors	51 (58.0)	7 (29.2)	0.023*
	Survivors	37 (42.0)	17 (70.8)	

*Pearson's chi-square test. ** Yates' corrected chi-square test. *** Fisher's exact test.

Table S4: Comparison of categorical variables according to tumor location

Variables	Categories	Cardia	Corpus	Distal	p
Gender	Female	4 (14.3)	13 (44.8)	15 (27.3)	0.037
	Male	24 (85.7)	16 (55.2)	40 (72.7)	
Type of Resection	Total	25 (89.3)	18 (62.1)	14 (25.5)	<0.001
	Near Total	0 (0.0)	1 (3.4)	4 (7.3)	
	Subtotal	3 (10.7)	10 (34.5)	37 (67.3)	
Lauren Classification	Poorly cohesive	2 (7.1)	9 (31.0)	13 (23.6)	0.227
	Mixed	6 (21.4)	6 (20.7)	9 (16.4)	
	Intestinal	20 (71.4)	14 (48.3)	33 (60.0)	
WHO Classification	Poorly cohesive	2 (7.1)	9 (31.0)	13 (23.6)	0.439
	Mixed adenoca	6 (21.4)	6 (20.7)	9 (16.4)	
	Tubular adenoca	16 (57.1)	10 (34.5)	28 (50.9)	
	Papillary adenoca	4 (14.3)	3 (10.3)	4 (7.3)	
	Mucinous [illegible]	0 (0.0)	1 (3.4)	1 (1.8)	
Perineural Invasion	Present	19 (67.9)	21 (72.4)	36 (65.5)	0.810
	Absent	9 (32.1)	8 (27.6)	19 (34.5)	
Lymphovascular Invasion	Present	23 (82.1)	26 (89.7)	50 (90.9)	0.484
	Absent	5 (17.9)	3 (10.3)	5 (9.1)	
Tumor Stage	Advanced stage	21 (75.0)	24 (82.8)	43 (78.2)	0.771
	Early stage	7 (25.0)	5 (17.2)	12 (21.8)	
Pathological T Stage	T4b	2 (7.1)	6 (20.7)	5 (9.1)	0.681
	T4a	8 (28.6)	10 (34.5)	21 (38.2)	
	T3	11 (39.3)	8 (27.6)	17 (30.9)	
	T2	5 (17.9)	2 (6.9)	5 (9.1)	
	T1b	1 (3.6)	2 (6.9)	6 (10.9)	
	T1a	1 (3.6)	1 (3.4)	1 (1.8)	
Pathological N Stage	N3b	7 (25.0)	5 (17.2)	10 (18.2)	0.212
	N3a	4 (14.3)	13 (44.8)	17 (30.9)	
	N2	5 (17.9)	1 (3.4)	8 (14.5)	
	N1	5 (17.9)	5 (17.2)	14 (25.5)	
	N0	7 (25.0)	5 (17.2)	6 (10.9)	
Clinical M Stage	M1	4 (14.3)	9 (31.0)	12 (21.8)	0.313
	M0	24 (85.7)	20 (69.0)	43 (78.2)	
TNM Staging	IV	4 (14.3)	9 (31.0)	12 (21.8)	0.884
	IIIC	5 (17.9)	3 (10.3)	8 (14.5)	

	IIIB	3 (10.7)	7 (24.1)	7 (12.7)	
	IIIA	5 (17.9)	4 (13.8)	9 (16.4)	
	IIB	3 (10.7)	1 (3.4)	7 (12.7)	
	IIA	3 (10.7)	2 (6.9)	6 (10.9)	
	IB	3 (10.7)	1 (3.4)	3 (5.5)	
	IA	2 (7.1)	2 (6.9)	3 (5.5)	
Tumor Differentiation	Poorly	13 (46.4)	17 (58.6)	30 (54.5)	0.332
	Moderately	6 (21.4)	8 (27.6)	17 (30.9)	
	Well	9 (32.1)	4 (13.8)	8 (14.5)	
HER2/neu staining	Positive	7 (25.0)	4 (13.8)	4 (7.3)	0.085
	Negative	21 (75.0)	25 (86.2)	51 (92.7)	
Metastasis/ Recurrence	Present	7 (25.0)	12 (41.4)	14 (25.5)	0.263
	Absent	21 (75.0)	17 (58.6)	41 (74.5)	
Outcome	Non-survivors	14 (50.0)	20 (69.0)	24 (43.6)	0.085
	Survivors	14 (50.0)	9 (31.0)	31 (56.4)	

Table S5: Comparison of categorical variables according to metastasis status

Variables	Categories	Metastasis (+)	Metastasis (-)	p
Gender	Female	12 (36.4)	20 (25.3)	0.342**
	Male	21 (63.6)	59 (74.7)	
Tumor Location	Cardia	7 (21.2)	21 (26.6)	0.263*
	Corpus	12 (36.4)	17 (21.5)	
	Distal	14 (42.4)	41 (51.9)	
Type of Resection	Total	16 (48.5)	41 (51.9)	0.268*
	Near Total	0 (0.0)	5 (6.3)	
	Subtotal	17 (51.5)	33 (41.8)	
Lauren Classification	Poorly cohesive	9 (27.3)	15 (19.0)	0.488*
	Mixed	7 (21.2)	14 (17.7)	
	Intestinal	17 (51.5)	50 (63.3)	
WHO Classification	Poorly cohesive	9 (27.3)	15 (19.0)	0.416
	Mixed adenoca	7 (21.2)	14 (17.7)	
	Tubular adenoca	16 (48.5)	38 (48.1)	
	Papillary adenoca	1 (3.0)	10 (12.7)	
	Mucinous adenoca	0 (0.0)	2 (2.5)	
Perineural Invasion	Present	30 (90.9)	46 (58.2)	0.001**
	Absent	3 (9.1)	33 (41.8)	
Lymphovascular Invasion	Present	32 (97.0)	67 (84.8)	0.104***
	Absent	1 (3.0)	12 (15.2)	
Tumor Stage	Advanced stage	32 (97.0)	56 (70.9)	0.005**
	Early stage	1 (3.0)	23 (29.1)	
Pathological T Stage	T4b	7 (21.2)	6 (7.6)	0.003*
	T4a	18 (54.5)	21 (26.6)	
	T3	7 (21.2)	29 (36.7)	
	T2	1 (3.0)	11 (13.9)	
	T1b	0 (0.0)	9 (11.4)	
	T1a	0 (0.0)	3 (3.8)	
Pathological N Stage	N3b	12 (36.4)	10 (12.7)	0.013*
	N3a	11 (33.3)	23 (29.1)	
	N2	4 (12.1)	10 (12.7)	
	N1	5 (15.2)	19 (24.1)	
	N0	1 (3.0)	17 (21.5)	

TNM Staging	IV	16 (48.5)	9 (11.4)	0.001
	IIIC	4 (12.1)	12 (15.2)	
	IIIB	5 (15.2)	12 (15.2)	
	IIIA	6 (18.2)	12 (15.2)	
	IIB	1 (3.0)	10 (12.7)	
	IIA	1 (3.0)	10 (12.7)	
	IB	0 (0.0)	7 (8.9)	
	IA	0 (0.0)	7 (8.9)	
Tumor Differentiation	Poorly	22 (66.7)	38 (48.1)	0.134
	Moderately	8 (24.2)	23 (29.1)	
	Well	3 (9.1)	18 (22.8)	
HER2/neu staining	Positive	3 (9.1)	12 (15.2)	0.547***
	Negative	30 (90.9)	67 (84.8)	
Outcome	Non-survivors	29 (87.9)	29 (36.7)	<0.001**
	Survivors	4 (12.1)	50 (63.3)	

*Pearson's chi-square test. ** Yates' corrected chi-square test. *** Fisher's exact test.

Table S6: Comparison of categorical variables according to mortality status

Variables	Categories	Non-survivor	Survivor	p
Gender	Female	18 (31.0)	14 (25.9)	0.698**
	Male	40 (69.0)	40 (74.1)	
Tumor Location	Cardia	14 (24.1)	14 (25.9)	0.085*
	Corpus	20 (34.5)	9 (16.7)	
	Distal	24 (41.4)	31 (57.4)	
Type of Resection	Total	32 (55.2)	25 (46.3)	0.055*
	Near Total	0 (0.0)	5 (9.3)	
	Subtotal	26 (44.8)	24 (44.4)	
Lauren Classification	Poorly cohesive	16 (27.6)	8 (14.8)	0.229*
	Mixed	11 (19.0)	10 (18.5)	
	Intestinal	31 (53.4)	36 (66.7)	
WHO Classification	Poorly cohesive	16 (27.6)	8 (14.8)	0.249*
	Mixed adenoca	11 (19.0)	10 (18.5)	
	Tubular adenoca	27 (46.6)	27 (50.0)	
	Papillary adenoca	4 (6.9)	7 (13.0)	
	Mucinous adenoca	0 (0.0)	2 (3.7)	
Perineural Invasion	Present	48 (82.8)	28 (51.9)	0.001**
	Absent	10 (17.2)	26 (48.1)	
Lymphovascular Invasion	Present	54 (93.1)	45 (83.3)	0.188**
	Absent	4 (6.9)	9 (16.7)	
Tumor Stage	Advanced stage	51 (87.9)	37 (68.5)	0.023**
	Early stage	7 (12.1)	17 (31.5)	
Pathological T Stage	T4b	10 (17.2)	3 (5.6)	0.040*
	T4a	25 (43.1)	14 (25.9)	
	T3	16 (27.6)	20 (37.0)	
	T2	4 (6.9)	8 (14.8)	
	T1b	2 (3.4)	7 (13.0)	
	T1a	1 (1.7)	2 (3.7)	
Pathological N Stage	N3b	18 (31.0)	4 (7.4)	0.007*
	N3a	18 (31.0)	16 (29.6)	
	N2	8 (13.8)	6 (11.1)	
	N1	9 (15.5)	15 (27.8)	
	N0	5 (8.6)	13 (24.1)	
Clinical M Stage	M1	21 (36.2)	4 (7.4)	0.001**
	M0	37 (63.8)	50 (92.6)	

TNM Staging	IV	21 (36.2)	4 (7.4)	<0.001*
	IIIC	9 (15.5)	7 (13.0)	
	IIIB	7 (12.1)	10 (18.5)	
	IIIA	13 (22.4)	5 (9.3)	
	IIB	1 (1.7)	10 (18.5)	
	IIA	3 (5.2)	8 (14.8)	
	IB	1 (1.7)	6 (11.1)	
	IA	3 (5.2)	4 (7.4)	
Tumor Differentiation	Poorly	39 (67.2)	21 (38.9)	0.011*
	Moderately	11 (19.0)	20 (37.0)	
	Well	8 (13.8)	13 (24.1)	
HER2/neu staining	Positive	6 (10.3)	9 (16.7)	0.481
	Negative	52 (89.7)	45 (83.3)	
Metastasis/Recurrence	Present	29 (50.0)	4 (7.4)	<0.001**
	Absent	29 (50.0)	50 (92.6)	

*Pearson's chi-square test. ** Yates' corrected chi-square test. *** Fisher's exact test.

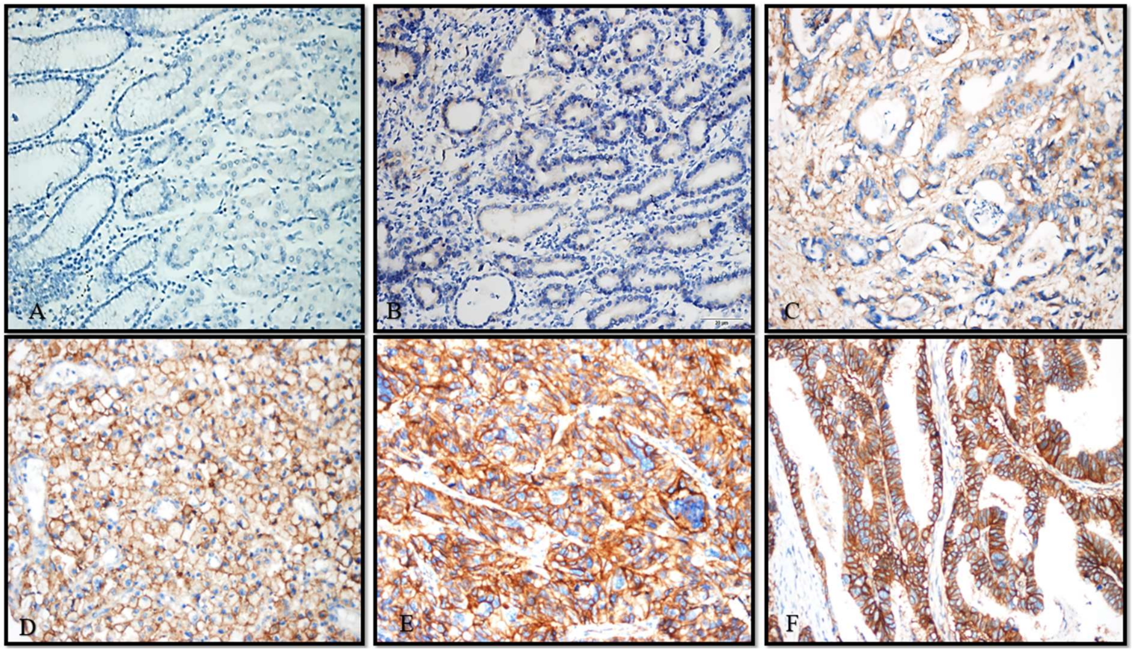

Figure S1: CD155 (PVR) expression in normal gastric tissue and gastric carcinoma ,CD155x200. A: Normal gastric tissue shows no CD 155 expression. B: Tubulary gastric carcinoma shows weak and moderate (C) CD155 expression. D-F: strong CD 155 expression in signet cell (D), poorly differentiated tubular (E), and papillary carcinoma (F).

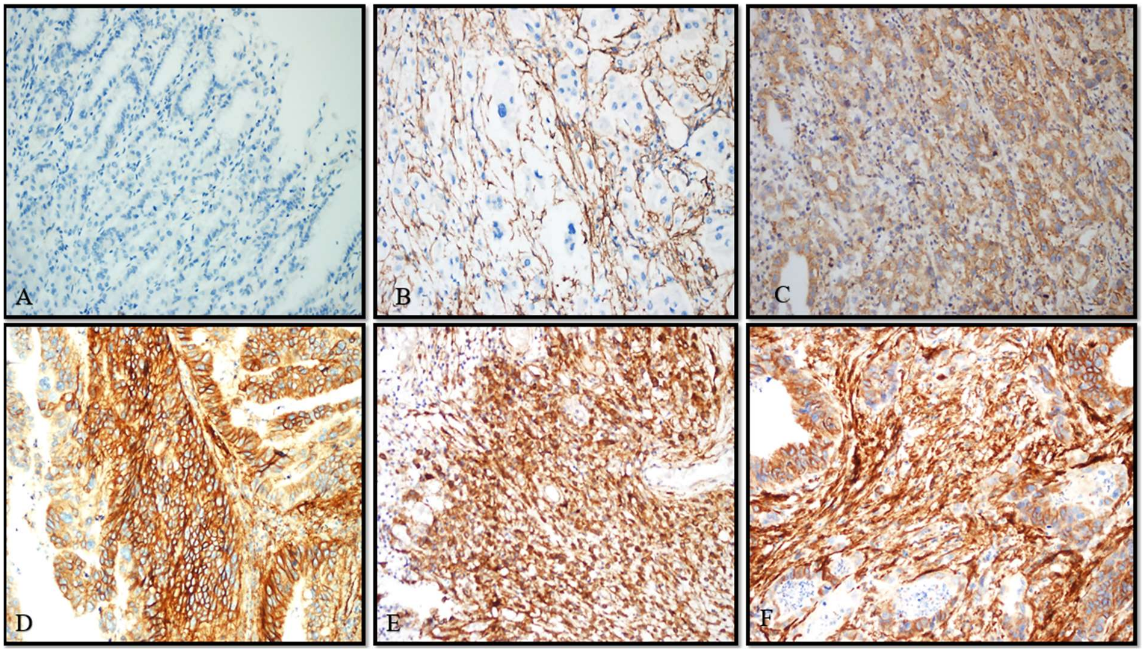

Figure S2: B7-H3 (CD276) expression in normal gastric tissue and gastric carcinoma, B7-H3x200. A: Normal gastric tissue shows no B7-H3 expression. B: Gastric carcinoma shows no expression in epithelial component whereas stromal cells show positive expression. C: Moderate tumoral epithelial expression in tubulary gastric carcinoma. D-E: Strong tumoral epithelial expression in gastric carcinoma. F: Strong epithelial and stromal expression in gastric carcinoma.
